# Cleaved CD31 as a target for *in vivo* molecular imaging of inflammation

**DOI:** 10.1038/s41598-019-56163-x

**Published:** 2019-12-20

**Authors:** Jonathan Vigne, Sylvie Bay, Rachida Aid-Launais, Guillaume Pariscoat, Guillaume Rucher, Jean Sénémaud, Ariane Truffier, Nadège Anizan, Guillaume Even, Christelle Ganneau, Francesco Andreata, Marie Le Borgne, Antonino Nicoletti, Dominique Le Guludec, Giuseppina Caligiuri, Francois Rouzet

**Affiliations:** 1 Nuclear Medicine Department, X. Bichat Hospital, APHP and DHU FIRE, F-75018 Paris, France; 2 Université de Paris, LVTS, INSERM U1148, F-75018 Paris, France; 3 Université de Paris, UMS34 FRIM, F-75018 Paris, France; 4 Unité Chimie des Biomolécules, Département Biologie Structurale et Chimie, Institut Pasteur, Paris, France; 5 CNRS UMR 3523, Institut Pasteur, Paris, France

**Keywords:** Diagnostic markers, Imaging the immune system

## Abstract

There is a need for new targets to specifically localize inflammatory foci, usable in a wide range of organs. Here, we hypothesized that the cleaved molecular form of CD31 is a suitable target for molecular imaging of inflammation. We evaluated a bioconjugate of D-P8RI, a synthetic peptide that binds all cells with cleaved CD31, in an experimental rat model of sterile acute inflammation. Male Wistar rats were injected with turpentine oil into the gastrocnemius muscle two days before ^99m^Tc-HYNIC-D-P8RI (or its analogue with L-Proline) SPECT/CT or [^18^F]FDG PET/MRI. Biodistribution, stability study, histology, imaging and autoradiography of ^99m^Tc-HYNIC-D-P8RI were further performed. Biodistribution studies revealed rapid elimination of ^99m^Tc-HYNIC-D-P8RI through renal excretion with almost no uptake from most organs and excellent *in vitro* and *in vivo* stability were observed. SPECT/CT imaging showed a significant higher ^99m^Tc-HYNIC-D-P8RI uptake compared with its analogue with L-Proline (negative control) and no significant difference compared with [^18^F]FDG (positive control). Moreover, autoradiography and histology revealed a co-localization between ^99m^Tc-HYNIC-D-P8RI uptake and inflammatory cell infiltration. ^99m^Tc-HYNIC-D-P8RI constitutes a new tool for the detection and localization of inflammatory sites. Our work suggests that targeting cleaved CD31 is an attractive strategy for the specific *in vivo* imaging of inflammatory processes.

## Introduction

Inflammatory disorders can affect virtually any organ and are a frequent cause of morbidity. Recently, immunotherapies emerged as highly efficient treatments in specific inflammatory diseases. Since their prescription is rapidly increasing, their cost represents a growing burden for healthcare systems. It is therefore highly desirable to guide the use of such expensive therapies by dedicated biomarkers. The high level of glucose consumption during inflammatory processes has prompted the use of 2-deoxy-2-[^18^F]fluoro-D-glucose ([^18^F]FDG) positron emission tomography (PET) in the management of a wide variety of inflammatory disorders^1^. [^18^F]FDG however suffers from poor specificity since glycolysis is not limited to aseptic inflammation but is also present in infection, cancer and their related inflammatory processes. Recently, the need to develop biomarkers targeting specifically the host immune response in cancer has emerged^2^. In addition, [^18^F]FDG is avidly taken up by organs such as brain or heart in physiological conditions thereby hampering its diagnostic value.

Inflammation involves surface expression of endothelial adhesion molecules, platelet adhesion and activation of leukocytes resulting in leukocyte transmigration into tissues^3–5^. An example of such adhesion molecules is the CD31 receptor also known as PECAM-1, a transmembrane homophilic receptor^6–8^ which is constitutively and exclusively expressed by endothelial cells, platelets and leukocytes. CD31 is highly concentrated at the intercellular junctions between endothelial cells^9–11^ and plays an important role in the homeostasis of the endothelial barrier function. In addition, CD31 plays a determinant role in the regulation of leukocyte and platelet activation^12–18^. The extracellular portion of CD31 is organized into six Ig-like domains. Domain 1 mediates the trans-homophilic CD31-CD31 binding when two CD31+ cells interact^19,20^. This trans-homophilic binding induces a cis-homodimerisation of CD31 juxta-membrane portion^19^ and triggers a specific inhibitory tyrosine phosphate-dependent signaling through the CD31 cytoplasmic domain. By these mechanisms, CD31 appears as a central regulator of inflammatory responses as it modulates the recruitment and extravasation of immune cells at sites of platelet-coated, inflamed microvessels^21^.

Importantly, the CD31-mediated regulation is determined by its molecular integrity. In pro-inflammatory conditions, the CD31 extracellular portion is cleaved so that recently activated cells express a truncated form of CD31 (cleaved CD31). The loss of the trans-homophilic domain of CD31 invalidates its inhibitory function and allows the full activation of cleaved CD31 cells^16,22^.

We hypothesized that the cleaved molecular form of CD31 might be a suitable target for molecular imaging because it is present in large amounts at the surface of the three major cell types at stake at sites of active inflammation (leukocytes, platelets and endothelial cells). To target cleaved CD31, we selected an octapeptide (H-RVFLAPWK-OH) derived from the immunosuppressive CD31 peptide^23^ and further engineered as the retro-inverso analogue, i.e. the reverse sequence with D-amino acids^24^. The resulting functional mimic of the parent peptide (H-kwpalfvr-OH) is a good ligand candidate because it is stable *in vivo* and it specifically binds to the cleaved cis-homophilic juxta-membrane sequence of the CD31 ectodomain (aa 551–574) common to all cleaved CD31 cells^22,24^.

Herein, we designed and prepared a bioconjugate of D-P8RI coupled to 6-Hydrazinonicotinic acid (HYNIC) as a bifunctional complexing agent for technetium-99m (^99m^Tc)^25^. We then assessed the performance of the resulting radiotracer ^99m^Tc-HYNIC-D-P8RI for *in vivo* non-invasive imaging of inflammatory cells in an experimental rat model comparatively to its counterpart with L-Proline (L-P8RI) and to the well-established radiolabeled glucose analogue 2-deoxy-2-[^18^F]fluoro-D-glucose ([^18^F]FDG) as controls.

## Results

### Design, synthesis and physico-chemical characterization of the Tc-labeled D-P8RI

**Figure 1:**
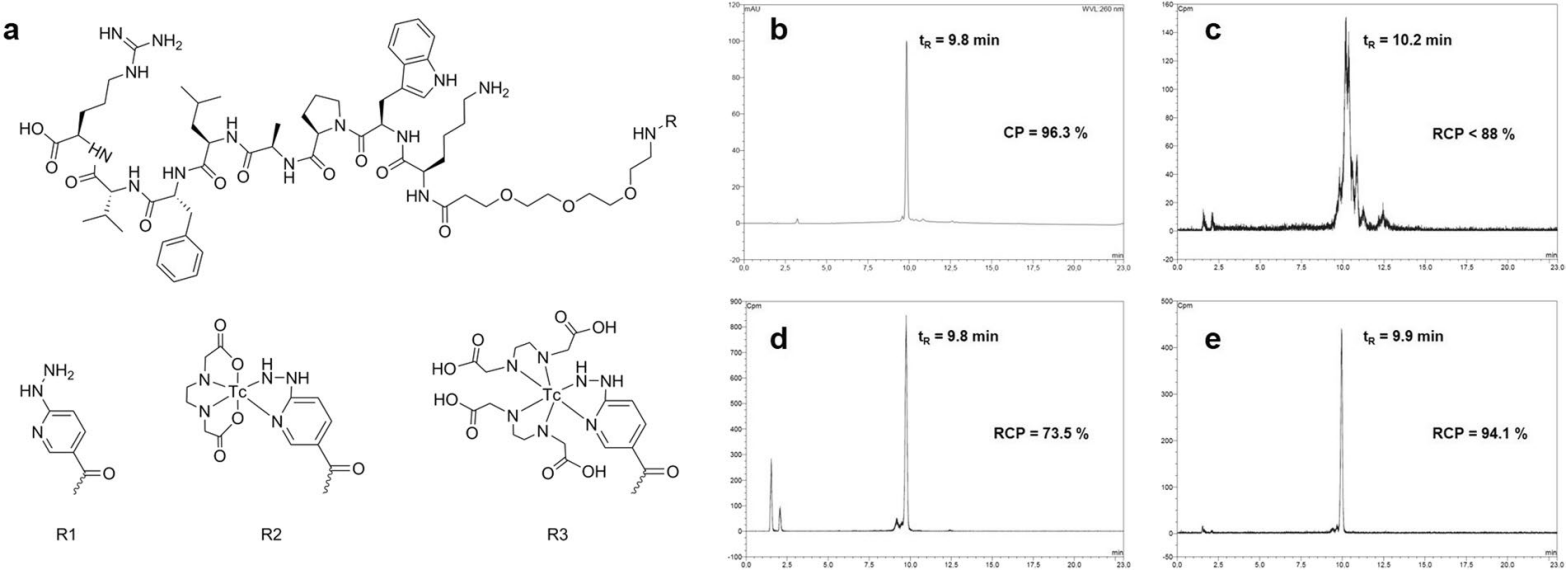
(**a**) P8RI-based conjugates: HYNIC-D-P8RI (R = R1) and putative structure of its corresponding EDDA complexes after labeling: [^99m^Tc(HYNIC-D-P8RI)(EDDA)] (R = R2) and [^99m^Tc(HYNIC-D-P8RI)(EDDA_2_)] (R = R3). (**b**–**e**) Optimization of the radiolabeling process. **b** RP-HPLC chromatograms of: HYNIC-D-P8RI (UV detection); (**c**–**e**) ^99m^Tc-HYNIC-D-P8RI (radio detection) using tricine as coligand, EDDA as coligand and tricine/EDDA exchange strategy, respectively. The chemical or radiochemical purity (CP or RCP), as well as the retention time (t_R_) are indicated on each panel.

The bioconjugate radiotracer is composed of (i) the D-P8RI peptide sequence which targets the cleaved CD31 molecular species, (ii) the HYNIC group enabling ^99m^Tc chelation, (iii) a PEG linker between both moieties to reduce steric hindrance, preserve the P8RI binding capacity and maintain hydrophilic properties, and iv) the radioisotope ^99m^Tc for SPECT imaging. Results of the synthesis of HYNIC-D-P8RI are detailed in Supplementary Material (see Fig. 1a (R = R1) for structural formula, and Fig. 1b for RP-HPLC profile).

This peptide derivative was further labeled using different coligands. The profile obtained with tricine was heterogenous and the RCP was <88% as assessed by RP-HPLC (Fig. 1c) whereas EDDA and tricine/EDDA exchange labeling yielded a dominant radiolabeled species profile with RCP <74% and ≥94%, respectively (Fig. 1d,e). Retention factors (Rf) of radiolabeled HYNIC-D-P8RI evaluated with TLC in the different mobile phases were: Rf = 0 in methylethylketone (MEK) and Anticoagulant Citrate Dextrose Solution (ACD), Rf = 0.8–1 in CH_3_CN/H_2_O (3/2) allowing to estimate radiolabeling yields at 92.6, 71.1, and 95.3% for tricine, EDDA and tricine/EDDA experiments, respectively (data not shown). Radiolabeling impurities found in TLC corresponded to non-peptide bound species such as ^99m^Tc-coligands and ^99m^TcO4^−^, no ^99m^Tc-colloid was detected. The tricine/EDDA exchange labeling strategy was chosen for the following experiments based on the high yield, on the high RCP obtained and the high specific activity over 114 GBq/µmoL.

To identify the coordination state of the complex by MS, ^99^Tc-HYNIC-D-P8RI was prepared using the tricine/EDDA process except that ^99m^Tc was replaced by a larger amount of ^99^Tc. A summary of HPLC/MS analyses is presented (Table 1). When compared with the control unlabeled peptide, the mass spectrum of the labeled conjugate showed an increase of 270 Da and 446 Da, which can be assigned to coexisting Tc complexes coordinated with one and two EDDA, respectively (Fig. 1a, R = R2, R3). Peptide species containing tricine were not present to any significant degree.

**Table 1:** Selected HPLC/MS data for HYNIC-D-P8RI and its corresponding Tc conjugates. Calculated *m/z* values are based on the exact molecular mass using MassLynx calculator (Waters, France). M refers to the unlabeled and uncharged peptide conjugate HYNIC-P8RI.

Compound	*m/z* calculated	*m/z* observed	Assignment
HYNIC-D-P8RI	1354.764	1354.718	[M + H]^+^
[^99^Tc(HYNIC-D-P8RI)(EDDA)]	1624.750	1625.116	[(M + ^99^Tc + EDDA - 5 H) + H]^+^
[^99^Tc(HYNIC-D-P8RI)(EDDA_2_)]	1800.830	1801.226	[(M + ^99^Tc + 2 EDDA - 5 H) + H]^+^

### *In vitro* properties

**Figure 2:**
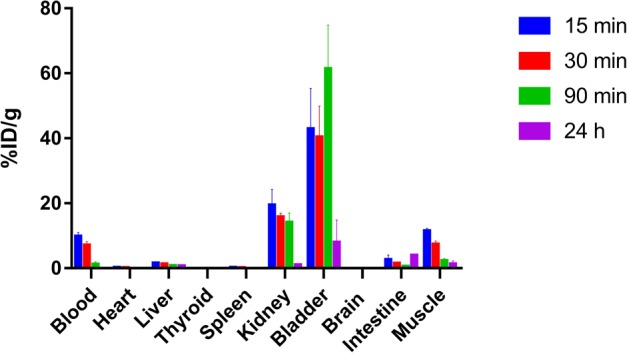
*In vitro* assessment of ^99m^Tc-HYNIC-D-P8RI in human plasma (**a**,**b**). (**a**) Percentage of human plasma protein binding over 4 h (mean ± SD) evaluated by size exclusion chromatography. Control (grey bar) refers to the same experiment performed in PBS instead of human plasma. (**b**) Percentage of intact ^99m^Tc-HYNIC-D-P8RI in human plasma over 4 h, referred as RCP and assessed by RP-HPLC. (**c**) Biodistribution (%ID/g, n = 3) of ^99m^Tc-HYNIC-D-P8RI in male Wistar rats at 15, 30, 90 min and 24 h post-injection. Columns, mean; bars, SD. (**d**) RP-HPLC chromatogram (radio detection) of rat urine 90 min p.i; radiochemical purity (RCP) and retention time (t_R_) are indicated.

Percentage of ^99m^Tc-HYNIC-D-P8RI bound to plasma protein were: 7.16 ± 0.44, 4.80 ± 1.36, 3.75 ± 1.12, 4.84 ± 1.17, 5.68 ± 0.75, 5.83 ± 0.51 and 1.23 ± 0.55 for 0, 0.5, 1, 2, 3 and 4 h of incubation and control (plasma replaced by PBS), respectively (Fig. 2a). Percentage of intact ^99m^Tc-HYNIC-D-P8RI referring to plasma stability was: 93.02 ± 0.04, 91.05 ± 0.02, 89.07 ± 0.02 for 1, 2 and 3 h of incubation, respectively (Fig. 2b). The octanol-water partition coefficient Log P value was: −2.92 ± 0.04.

### *In vivo* properties

#### Biodistribution and *in vivo* stability

Results of biodistribution in animals at 15, 30, 90 min and 24 h p.i. are summarized in Fig. 2c. Almost no uptake from most organs and rapid clearance through renal excretion was observed. The accumulated activity was always <0.2% ID/g in heart, thyroid, spleen and brain. Urinary clearance was predominant (14.04 ± 2.93% ID/g for kidney and 61.38 ± 13.42% ID/g for bladder 90 min p.i.) concomitant to a rapid clearance from blood (9.74 ± 1.21% ID/g 15 min p.i and 0.04 ± 0.02% ID/g 24 h p.i). Based on biodistribution results, images acquisition in animal injected with Turpentine has been set 1 h p.i. because of the very low activity remaining in blood and muscle. Urine RP-HPLC exhibited a single radiolabeled species profile similar to the injected peptide (t_R_ = 10.0 min and RCP 93.1%) as shown in Fig. 2d.

#### SPECT/CT and PET/MRI imaging

**Figure 3:**
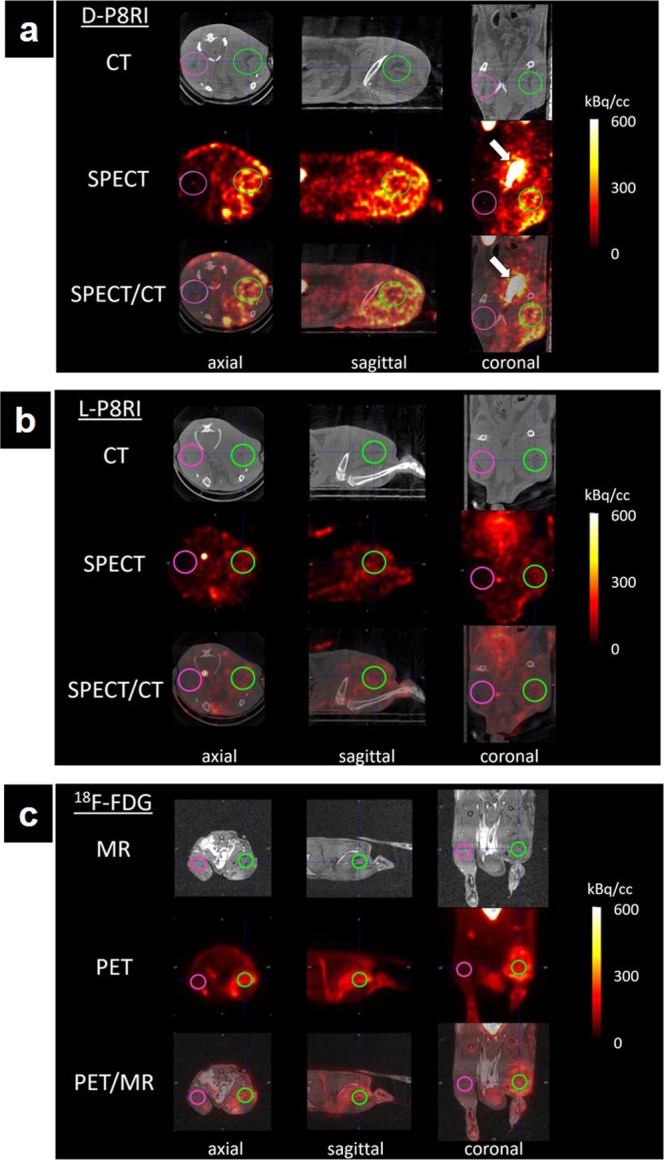
Axial, sagittal and coronal views of rats left (saline) and right (Turpentine oil) hind limbs (pink and green regions of interest, respectively) obtained with ^99m^Tc-HYNIC-D-P8RI (**a**), ^99m^Tc-HYNIC-L-P8RI (**b**) SPECT/CT, and [^18^F]FDG PET/MR (**c**). ^99m^Tc-HYNIC-D-P8RI and [^18^F]FDG uptake was associated with local inflammation compared with a low signal of ^99m^Tc-HYNIC-L-P8RI. All radiotracers displayed a very low uptake in the contralateral hindlimb (control). Bladder is pointed with a white arrow in (**a**).

**Figure 4:**
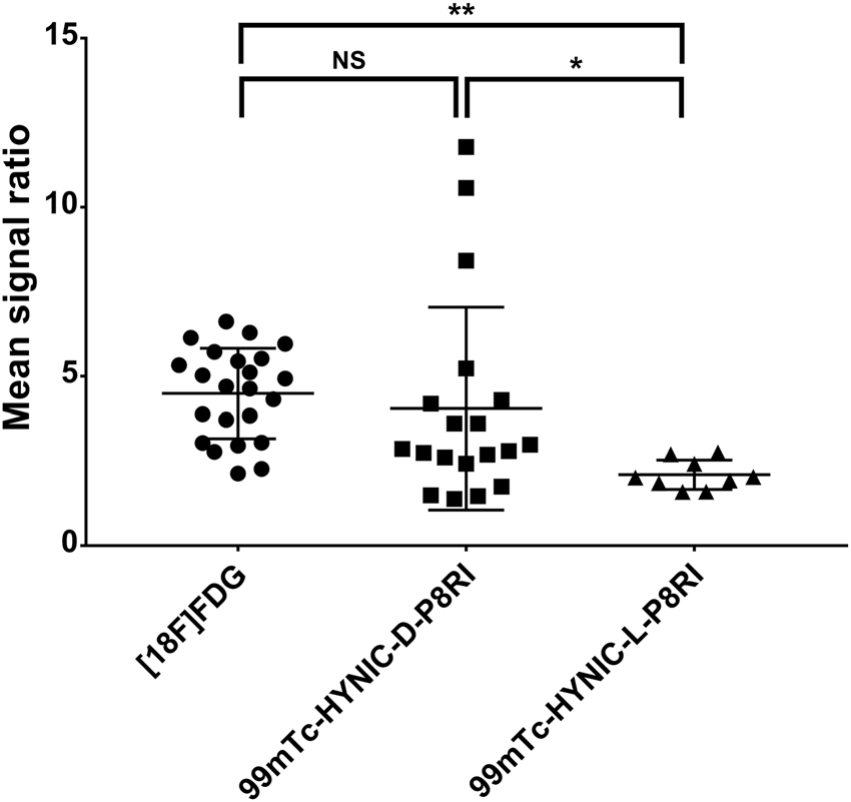
Mean signal ratios (Mean signal inflammatory muscle/Mean signal contralateral muscle) calculated on scintigrams from the [^18^F]FDG, ^99m^Tc-HYNIC-D-P8RI and ^99m^Tc-HYNIC-L-P8RI injected animals. *p < 0.05, **p < 0.01, NS: Non significant.

SPECT/CT and PET/MRI mainly showed a diffuse uptake of the different radiotracers in the turpentine injected hindlimb (TIH) that was more visible compared to the contralateral saline injected hindlimb (SIH) (Fig. 3). Mean signal ratios (TIH/SIH) were 4.49 ± 0.43 (n = 23), 4.05 ± 0.47 (n = 19) and 2.10 ± 0.68 (n = 9) for [^18^F]FDG, ^99m^Tc-HYNIC-D-P8RI and ^99m^Tc-HYNIC-L-P8RI, respectively (Fig. 4). One way Anova (F Ratio = 4.49, p < 0.02) followed by means comparison exhibited a significantly different mean signal ratio between: [^18^F]FDG and ^99m^Tc-HYNIC-L-P8RI (p < 0.01), ^99m^Tc-HYNIC-D-P8RI and ^99m^Tc-HYNIC-L-P8RI (p < 0.03) but not between [^18^F]FDG and ^99m^Tc-HYNIC-D-P8RI (p > 0.48).

#### Histology and autoradiography

**Figure 5:**
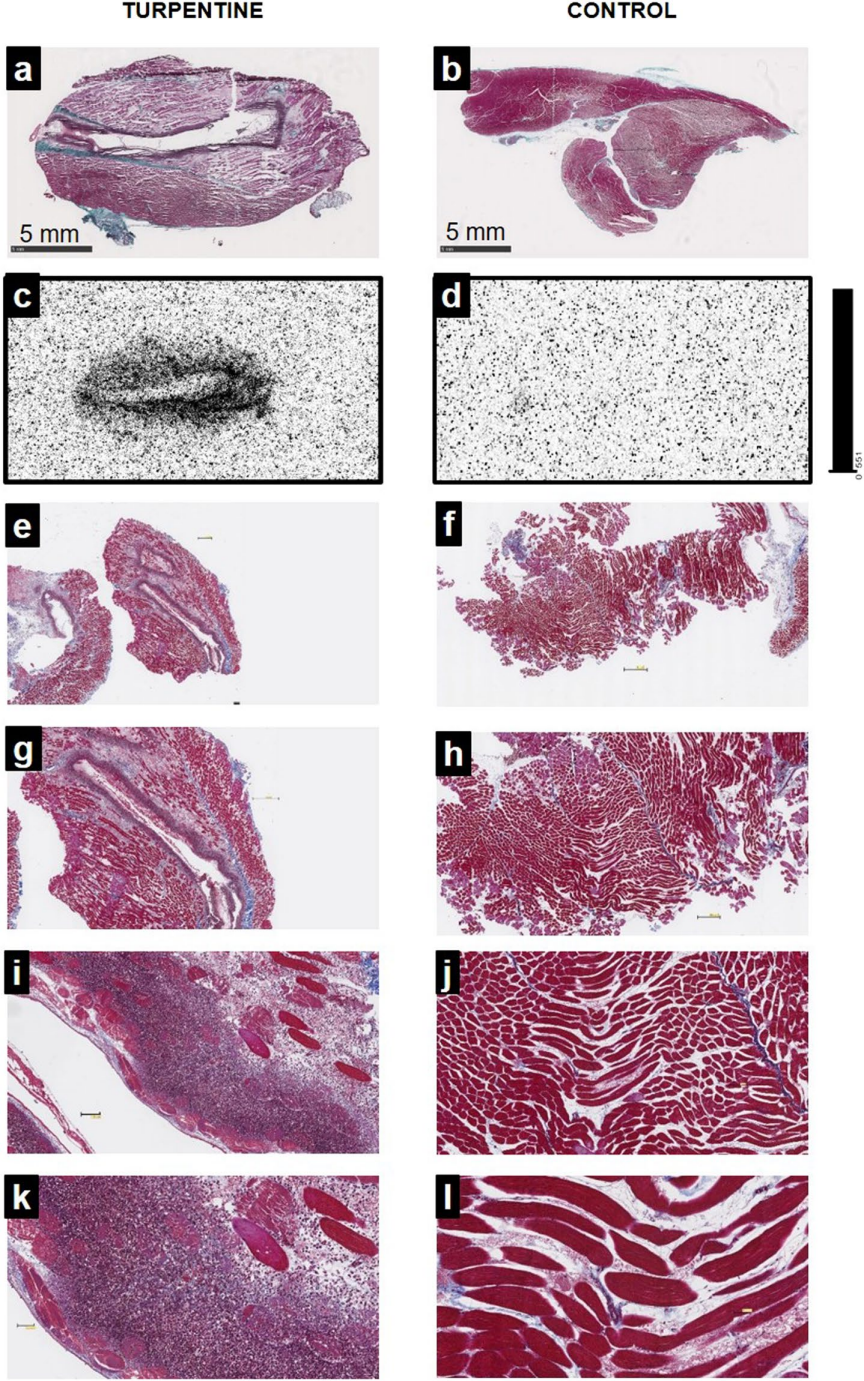
Masson’s trichrome staining (**a,b,e**–**l**) and autoradiography (**c**,**d**) of gastrocnemius muscles 2 days after intramuscular injection of saline (control, left hind limb) or turpentine oil (right hind limb). Animals were sacrificed 1 hour after intravenous ^99m^Tc-HYNIC-D-P8RI administration. Masson’s trichrome staining in (**a**) and (**b**) corresponds to the same slices of autoradiography in (**c**) and (**d**). Turpentine injected muscle (**a)** displays evidence of inflammation with edema and major leukocyte infiltration (**e,g,i,k**: x1.25, x2.5, x10, x20 respectively), co-localized with a high uptake of ^99m^Tc-HYNIC-D-P8RI. The representative micrographs (**b**) corresponds to a control muscle matching with a low uptake of ^99m^Tc-HYNIC-D-P8RI (**d**). Higher magnifications of the control show no evidence of inflammation (**f,h,j,l)**.

**Figure 6:**
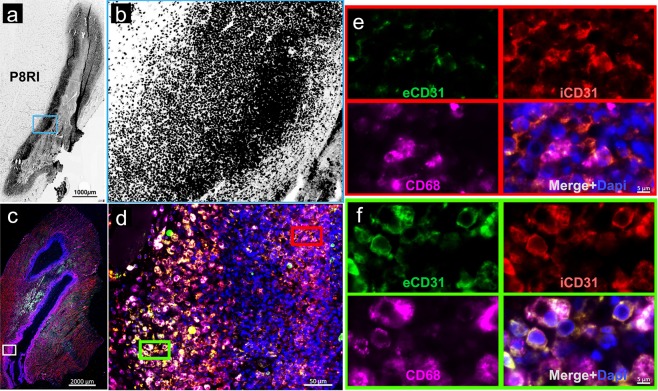
Fluorescence microscopy of the rat right gastrocnemius muscle, 2 days after turpentine oil injection. (**a**) Rhodamine labeled D-P8RI positive signal (black) is concentrated at the border of the turpentine oil injection. (**b**) Higher magnification in the blue inset reveals that D-P8RI labeled cells are enriched at the immediate vicinity of the damaged tissue. (**c**) Immunofluorescence of another rat right gastrocnemius muscle after turpentine oil injection. (**d**) The inner layer of the cells concentrated around the site of turpentine oil injection is mainly composed of CD68+ leucocytes (Fuchsia). (**e**) The mononuclear and polynuclear leukocytes (identified by the DAPI staining) at the immediate vicinity of the damaged tissue (red inset) display a cleavage of CD31 as revealed by the relative negative extracellular CD31 (eCD31) signal (green) in spite of a consistent intracellular CD31 (iCD31) staining (red). (**f**) The cleavage of CD31 was instead rather limited at the surface of the leukocytes located at the periphery of the inflammatory site (green inset). Of note, in parallel with a lesser CD31 cleavage phenomenon, the D-P8RI signal appears weaker in such an outer layer of the inflammatory site, as documented in (**b**).

Masson’s trichrome staining of the rat right gastrocnemius muscle, two days after turpentine injection, was characterized by a massive infiltration of leukocytes as compared with the preserved left muscles injected with saline (Fig. 5). Autoradiography of the same samples allowed to co-localize ^99m^Tc-HYNIC-D-P8RI uptake and inflammatory cell infiltration (Fig. 5c). Conversely, uptake in the contralateral muscle was low (Fig. 5d). This result was partially verified using rhodamine labelled D-P8RI which accumulated at the border of the turpentine oil injection site (Fig. 6a,b) on cells mainly composed of CD68+ leucocytes at the immediate vicinity of the damaged tissue (Fig. 6c,d).

Remarkably, in tissue-infiltrated leukocytes, the amount of extracellular CD31 was lower than that of intracellular CD31 (Fig. 6e,f), pointing at the cleavage of the molecule on these activated cells.

## Discussion

In the present work, we developed a completely novel approach for inflammation-specific imaging based on a synthetic peptide ligand targeting cleaved CD31 that is expressed upon activation by both inflammatory and endothelial cells. ^99m^Tc-HYNIC-D-P8RI was stable *in vivo* and allowed to detect acute sterile inflammation with a high specificity. Histological analysis proved the co-localization of ^99m^Tc-HYNIC-D-P8RI signal and leukocyte accumulation in tissues and suggested that the labeling is indeed mediated by cleaved CD31.

Initially considered as a cell adhesion molecule, CD31 is actually highly expressed at endothelial intercellular borders where it especially prevents leukocyte extravasation^26^. The native (non cleaved) CD31 is constitutively and exclusively expressed by platelets, leukocytes and endothelial cells at high levels (approximately one million copies of CD31 are present on the surface of endothelial cells)^27^. When native CD31 is engaged on interacting cells, it delivers a mutual ‘leave-me alone’ signal that mute cell activation^28^. However, when the activating stimuli is strong enough to overcome the activation threshold set by CD31, the function of the latter is invalidated by a proteolytic cleavage to allow full cell activation. The cleavage of CD31 occurs close to the cell membrane but leaves a small sequence of the truncated molecule lingering at the surface of recently activated cells. The D-P8RI specifically binds to this truncated molecular species and not to full length CD31. Since CD31 is encoded by a constitutive gene, in the absence of further stimulation, full CD31 molecules comes back within 3 hours at the surface of cells returning to baseline, to which P8RI cannot bind. Therefore, molecular imaging based on P8RI-mediated targeting of cleaved CD31 represents a relevant strategy to localize ongoing inflammatory processes.

The sequence of D-P8RI^24^ (patent WO2013190014A1) was designed based on the results of a functional screening of a large library of peptides derived from the parent 23 aa sequence corresponding to the epitope of the LYP21 antibody (aa 551–574), located within the cleaved cis-homophilic juxta-membrane sequence of the CD31 ectodomain^22,23^. Herein, we described the method to modify the P8RI sequence at the alpha position of the N-ter D-Lysine. A previous study has shown that N-ter modification of a P8RI containing peptide does not adversely affect its properties and inhibits lymphocyte activation *in vitro* and *in vivo*^22^. Also, D-P8RI was designed as a retro-inverso peptide with all-D-amino acids allowing to maintain bioactivity and to confer resistance towards plasma proteases^24,29,30^. Indeed, urine RP-HPLC analysis demonstrated that the tracer is eliminated unchanged indicating that most of it is not subjected to a catabolic degradation.

Quite a big spread in the mean signal ratio of ^99m^Tc-HYNIC-D-P8RI injected animals was observed. This may be explained by the model variability and especially difficulty to control turpentine oil diffusion at the site of injection which could be verified by a biodistribution study on turpentine oil injected animals. Also,the small size of the imaging agent is likely to facilitate diffusion in inflamed tissues. This feature ensures high sensitivity but may lead to decreased specificity because of possible non-specific retention of the tracer. To address this issue, we compared the signals derived from ^99m^Tc-HYNIC-D-P8RI and from its counterpart with L-Proline as a control. We showed that ^99m^Tc-HYNIC-D-P8RI had a significantly higher uptake in the inflammatory area compared to ^99m^Tc-HYNIC-L-P8RI, suggesting signal specificity.

On the other hand, capillary permeability remains limited during early steps of acute inflammation or inflammation at a chronic stage. The wide spectrum of P8RI that includes binding sites on activated endothelium is likely to provide a high number of targets without the need to cross the capillary wall. *In vitro* and *in vivo* biodistribution assessment of ^99m^Tc-HYNIC-D-P8RI showed the absence of significant uptake in tissues other than those related to urinary elimination. Compared to other imaging agents such as [^18^F]FDG which is taken up by brain and myocardium, this absence of physiological background activity in most tissues is expected to improve the contrast of the signal. Taken together, these characteristics enable to anticipate a good sensitivity of detection in a wide range of clinical settings. In addition the uptake of ^99m^Tc-HYNIC-D-P8RI in our acute model of aseptic inflammation was comparable to that obtained with the well validated [^18^F]FDG.

Currently, [^18^F]FDG PET/CT is the modality of choice for the clinical diagnosis of inflammatory diseases and the treatment monitoring. The limitations related to such an approach relying on glycolytic activity are well acknowledged regarding both the lack of specificity and the lack of signal relevance in brain and to some extent in the myocardium.

^99m^Tc-HYNIC-D-P8RI presents interesting features to support future developments for translational imaging research such as: specific targeting, favorable biodistribution, *in vivo* stability, relatively straightforward production process and radiolabeling procedure and swift blood clearance that make the tracer suitable for early imaging with good contrast in a wide variety of indications, including those in which [^18^F]FDG is not optimal. In particular, D-P8RI may be an interesting tool to develop molecular imaging probes for the longitudinal monitoring of therapies. For instance, it may be suitable in the field of autoimmune diseases in which [^18^F]FDG failed to reliably monitor inflammation such as arthritis in which there is a need to select patients that could be eligible for innovative anti-inflammatory immunotherapies^31,32^.

## Conclusions

^99m^Tc-HYNIC-D-P8RI constitutes a new tool for the detection and localization of inflammatory sites. Our work suggests that targeting cleaved CD31 is an attractive strategy for the specific *in vivo* imaging of inflammatory cells.

## Methods

Three different radiotracers were used in this study. First, ^99m^Tc-HYNIC-D-P8RI, the candidate radiotracer was prepared and evaluated. Second, its homologue with L-Proline, ^99m^Tc-HYNIC-L-P8RI, was used as a control and dedicated to target specificity evaluation. Finally, [^18^F]FDG was employed as a positive control.

Reagents were purchased from Sigma/Aldrich (St. Louis, MO, USA), except when otherwise stated. HYNIC-P8RI synthesis, reverse-phase (RP) high performance liquid chromatography (HPLC), electrospray ionization (ESI) mass spectrometry (MS), chemical and radiochemical purity (CP and RCP), thin layer chromatography (TLC), Log P value and fluorescence microscopy are available in Supplementary Data.

### Radiotracers

[^18^F]FDG was obtained from Advanced Accelerator Applications (Saint Genis Pouilly, France) and used as received. HYNIC-D-P8RI and HYNIC-L-P8RI were synthesized in-house (as described in Supplementary Data).

### Radiolabeling

In order to select the optimal radiolabeling approach, ^99m^Tc labeling of HYNIC-D-P8RI was assessed using different co-ligands: 1/Tricine as coligand: in a rubber-sealed N_2_ purged vial, HYNIC-D-P8RI (20 µg) was incubated with a tricine solution (250 μL, 40 mg/mL in PBS buffer pH 7.2), tin(II) chloride dihydrate solution (20 μL, 1 mg/mL in 0.1 N HCl), 1 GBq sodium pertechnetate (Na^99m^TcO4) and saline for a total volume of 1 mL. The mixture was incubated for 30 min at room temperature (RT); 2/Ethylenediamine-N,N′-diacetic acid (EDDA) as coligand: the tricine procedure was followed but with EDDA (250 µL, 20 mg/mL in 0.1 N NaOH) instead of tricine; 3/Tricine-EDDA coligands exchange labeling: the tricine procedure was followed except that EDDA (250 µL, 20 mg/mL in 0.1 N NaOH) was added to the reaction vial. The mixture was then incubated for 10 min at 100 °C.

The radiochemical purity (RCP) was determined as described in the Supplementary Material.

### Protein binding

Protein binding of the purified radiolabeled D-P8RI peptide (final concentration 100 pmol/mL) was determined in triplicate after 0, 0.5, 1, 2, 3 and 4 h of incubation in fresh human plasma (collected in citrate tubes) at 37 °C and analyzed after size exclusion chromatography (illustra Microspin G-50 Columns, Sephadex G-50, GE Healthcare, UK). Simultaneously, radiolabeled peptide was incubated in PBS at 37 °C as a control. G-50 columns were prespun at 2000 × g for 1 min then 20 μL of mixture was added and the column was centrifuged at 2000 × g for 2 min. Columns and eluates were counted on a Wallac Wizard 3″ 1480 (Perkin Elmer, MA, USA), then protein binding was calculated using the following activity (A) ratio: A eluate/(A column + A eluate).

### Plasma stability *in vitro*

Plasma stability was assessed in triplicate at 1, 2 and 4 h after incubation of the radiolabeled D-P8RI peptide (final concentration 100 pmol/mL) in human plasma at 37 °C with RCP ≥94%. After precipitation of plasma proteins with acetonitrile and centrifugation (15000 × g, 10 min), the supernatant was analyzed by RP-HPLC as described in Supplementary Data. Plasma stability was expressed in RCP (%).

### Animal model

Two to 3-month-old adult male Wistar rats, weighing 250–300 g were used. The animals were housed in a controlled environment. The procedures and animal care complied with the principles formulated by the National Society for Medical Research. All methods were performed in accordance with the guidelines of the French Directorate of Veterinary Services (authorization: No. 75-214). All methods were approved by the animal ethics committee of the Claude Bernard Institute (APAFIS #14821, Paris, France). Aseptic inflammation was obtained according to a standardized procedure consisting of turpentine oil injection characterized by a massive local influx of activated inflammatory cells^33,34^. In all *in vivo* imaging experiments, animals were treated with both 0.15 mL of turpentine by intramuscular (I.M.) injection into the right gastrocnemius muscle (hind-limb) and 0.15 mL of sterile saline in the left contralateral muscle.

### Biodistribution and *in vivo* stability

Biodistribution of ^99m^Tc-HYNIC-D-P8RI was performed 15, 30, 90 min and 24 h post-injection. Animals (n = 3 for each time point) were injected intravenously through penis vein with 30 ± 3 MBq of ^99m^Tc-HYNIC-D-P8RI. They were sacrificed by isoflurane overdose and blood, heart, liver, thyroid, spleen, kidney, brain, intestine and muscle tissues were harvested and counted using a well counter. Results were expressed as the percentage of injected dose per gram of tissue (% ID/g). To characterize radiotracer elimination and potential degradation, rat urine was collected 90 min p.i, passed through a 0.22 µm PVDF filter (Durapore^TM^ 13 mm, Merck), and analyzed using RP-HPLC as described in Supplementary Data.

### *In vivo* imaging procedures

Imaging procedures of ^99m^Tc-HYNIC-P8RI (D and L) were performed using Single Photon Emission Computed Tomography coupled to X-ray computed tomography (SPECT/CT). Acquisitions were performed two days after turpentine injection, on a dedicated small-animal hybrid camera (NanoSPECT/CT, Mediso, Hungary) equipped with multi-pinhole high-resolution collimators, (256 × 256 matrix), and a 20% energy window centered on 140 keV. *In vivo* scintigraphy was performed 1 h post intravenous injection (p.i.) through the penis vein of 74 ± 7 MBq of ^99m^Tc-HYNIC-P8RI under isoflurane anesthesia. Prior to scintigraphic acquisition, a whole-body high-resolution CT scan (45 keV/145 mAs; matrix size 256 × 256) was performed for anatomic localization. Iterative reconstruction of SPECT acquisitions was performed using Tera-Tomo software (Mediso) with ordered-subsets expectation maximization (4 iterations, 8 subsets) and Butterworth 3-dimensional post-filtering (cutoff frequency, 1.3 cycle/pixel; order, 5). Reconstructed slices were visually assessed in 3 planes (axial, coronal, and sagittal).

[^18^F]FDG PET/MRI scans were acquired using a Mediso NanoScan PET/MRI (1 T MRI, Mediso Ltd., Budapest, Hungary). After retro-orbital injection of the radiotracer (≈20 MBq; 100–150 μL), PET acquisitions were performed 1 h post-injection (p.i.) followed by MRI acquisition for anatomic localization. PET data were reconstructed using Tera-Tomo™ 3D software (Mediso Ltd., Budapest, Hungary), a 3D-OSEM Monte Carlo based algorithm with attenuation and scattering corrections (voxel size equal to 0.3 × 0.3 × 0.3 mm^3^). Intensity value in the voxels was calibrated in kilo Becquerel per cubic centimeter (kBq.cm-3) corrected for decay and positron branching ratio of ^18^F.

### Imaging data analysis

Three-dimensional Regions of Interests (3D-ROIs) were placed on the inflammatory muscles and the mean signal per volume unit (kBq/mL) was measured. Assuming a tissue density of 1 g/mL, the radioactivity contained in the ROI was divided by the dose administered to the animal and the volume of the ROI to obtain an imaging ROI-derived % ID/g. The same ROI was applied to the contralateral control muscle. The mean signal ratio was then calculated by dividing the mean signal from the inflammatory ROI by the mean signal from the contralateral ROI.

### *Ex vivo* quantification of ^99m^Tc-HYNIC-P8RI uptake

Autoradiography. TURP rats injected with ^99m^Tc-HYNIC-P8RI (D and L series) or [^18^F]FDG (74 MBq) were sacrificed 1 h post-injection. The posterior hindlimbs muscles were dissected, harvested en bloc and quickly frozen in OCT (Sakura, Torrance, CA, USA) at −80 °C for 15 min. A Leica cryomicrotome (Leica Microsystems Inc, Buffalo Grove, IL, USA) was used to obtain 20 μm-thick sections for autoradiography. ^99m^Tc-HYNIC-P8RI uptake on tissue slices was quantified using a calibrated bio-image analyzer CR-35 Bio (Elysia-Raytest, Liège, Belgium) after exposing sections for 24 h on dedicated phosphor imaging plates.

### Histology and fluorescence microscopy

Masson’s trichrome staining was performed on 10 µm-thick cryosections. Slices were air dried then fixed on Bouin’s fixative. Then slices were digitalized with a slide scanner (Nanozoomer 2.0RS, Hamamatsu, Japan) to correlate radiotracer uptake on autoradiography with anatomical landmarks. Immunohistochemistry was performed on paraffin embedded samples using: mouse anti rat CD68 monoclonal antibody (Antibody #MCA341R from Bio-Rad) and DAPI staining to identify mononuclear and polynuclear leukocytes. CD31 cleavage was evaluated using an anti-extracellular CD31 antibody (goat anti-rat antibody, #AF3628 from R&D systems) and an anti-intracellular CD31 antibody (rabbit anti-human antibody, #119-15418 from RayBiotech). Fluorescent tracking of D-P8RI was performed immediately before the sample harvesting using rhodamine labeled D-P8RI peptide injected through the left ventricular apex (bolus, 5 mg/kg) followed by perfusion of 10 mL saline to wash out all unbound peptide.

### Statistical *a*nalysis

Continuous variables were expressed as mean ± standard deviation and compared by use of one way ANOVA test followed by a post-hoc analysis (3 groups). Statistical significance between experimental groups was assessed using the JMP software v14.3 (SAS institute, France) with 95% confidence level. A value of p < 0.05 was considered significant.

## Supplementary information

DATASET 1
