# Supplementary material for: Cleaved CD31 as a target for in vivo molecular imaging of inflammation
Source: Sci Rep. 2019 Dec 20;9:19560. doi: 10.1038/s41598-019-56163-x (PMC6925130; doi:10.1038/s41598-019-56163-x)
Supplement: Supplementary file 1 — DATASET 1 [file 41598_2019_56163_MOESM1_ESM.docx]

**Cleaved CD31 as a target for *in vivo* molecular imaging of inflammation**

Jonathan Vigne ^1,2,3,^*****, Sylvie Bay ^4,5^ , Rachida Aid-Launais ^2,3^, Guillaume Pariscoat ^1,2,3^ , Guillaume Rucher ^2,3^, Jean Sénemaud ^3^, Ariane Truffier ^4^, Nadège Anizan ^2,3^, Guillaume Even ^2^, Christelle Ganneau ^4,5^, Francesco Andreata ^2^, Marie Le Borgne ^2^, Antonino Nicoletti ^2^, Dominique Le Guludec ^1,2,3^, Giuseppina Caligiuri ^2^, Francois Rouzet ^1,2,3^

^1^ Nuclear Medicine Department, X. Bichat Hospital, APHP and DHU FIRE, F-75018 Paris, France

^2^ Université de Paris, LVTS, INSERM U1148, F-75018 Paris, France.

^3^ Université de Paris, UMS34 FRIM, F-75018 Paris, France

^4^ Unité Chimie des Biomolécules, Département Biologie Structurale et Chimie, Institut Pasteur, Paris, France

^5^ CNRS UMR 3523, Institut Pasteur, Paris, France

**Correspondance:**

Dr J. Vigne

Service de Médecine Nucléaire

CHU Bichat, 46 rue Henri Huchard, 75018 PARIS, FRANCE

Tel: +33623203474

Fax: +33231064927

E-mail: [jonathan.vigne.fr@gmail.com](mailto:jonathan.vigne.fr@gmail.com)

Jonathan Vigne (PhD student) and Sylvie Bay contributed equally to the work

Giuseppina Caligiuri and Francois Rouzet contributed equally to the work

**SUPPLEMENTARY MATERIAL**

**MATERIALS & METHODS**

**HYNIC-P8RI synthesis**

HYNIC-D-P8RI was synthesized manually by solid phase peptide methodology using 9-fluorenylmethoxycarbonyl (Fmoc) chemistry, dimethylformamide (DMF) as solvent and 2-(1*H*-9-azabenzotriazole-1-yl)-1,1,3,3-tetramethyluronium hexafluorophosphate / *N*,*N*-diisopropylethylamine as the coupling reagents. Fmoc protection was removed with 20% piperidine in DMF. The octapeptide sequence was first assembled on a preloaded Fmoc-D-Arg(Pbf)-Wang resin (Merck, Germany) with all D-amino acids. After the last Fmoc deprotection, the peptide was further elongated in alpha position with 12-(Fmoc amino)-4,7,10-trioxa-dodecanoic acid (Iris Biotech, Germany) and 6-[2-(*tert*-Butoxycarbonyl)hydrazinyl]nicotinic acid (Interchim, France) in two cycles. After cleavage from the resin and deprotection in trifluoroacetic acid (TFA)/triisopropyl silane/H_2_O (95/2.5/2.5) during 30 min, the crude product was purified by reverse-phase (RP) flash chromatography (linear gradient 23-50% of CH_3_CN+0.1% TFA in H_2_O+0.1%TFA over 40 min) giving HYNIC-D-P8RI (9 mg, 52% overall yield). The purified conjugate was characterized by RP-HPLC (linear gradient 23-50% of CH_3_CN+0.1% TFA in H_2_O+0.1%TFA over 20 min, using a Kromasil C18 column (5 μm, 100Å, 250 x 4.6 mm) (AIT, France)), by electrospray ionization mass spectrometry (ESI-MS) after direct infusion, and by amino acid analysis (AAA) using a Beckman 6300 analyzer (Beckman Coulter, CA, USA) after hydrolysis with 6 N HCl at 110°C for 20 h. RP-HPLC: retention time 9.85 min, chemical purity was 96.3 %. ESI-MS: *m/z* calcd for C66H99N17O14: 1354.764 [M+H]^+^, 1376.746 [M+Na]^+^; found 1354.798, 1376.777. AAA: Ala 1 (1), Arg 1.03 (1), Leu 1.03 (1), Lys 1.27 (1), Phe 1.03 (1), Pro 1.04 (1), Val 1.07 (1). HYNIC-L-P8RI was prepared with the same procedure except L-Proline was used (36 mg, 55% overall yield, RP-HPLC retention time 11.6 min, chemical purity 96.8 %). ESI-MS: *m/z* calcd for C66H99N17O14: 1354.764 [M+H]^+^, 1376.746 [M+Na]^+^; found 1354.735, 1376.724. AAA: Ala 1 (1), Arg 0.98 (1), Leu 0.98 (1), Lys 1.27 (1), Phe 1.0 (1), Pro 0.98 (1), Val 0.96 (1).

Mass Spectrometry (MS)

The MS analysis of both HYNIC-P8RI was recorded after direct infusion in a Q-TOFMICRO spectrometer (Micromass) with an ESI (positive mode) source (Waters, France). The source temperature and the desolvation temperature were maintained at 80 °C and 250 °C, respectively. The cone voltage was 10 V and the collision energy 10 V. The sample was dissolved at a concentration of 5 μM in H_2_O/CH_3_CN (1/1) containing 0.1% formic acid (FA).

The HPLC/MS analyses were performed on an Alliance model 2695 system coupled to the same mass spectrometer and to a model 2487 UV detector (220 nm). The samples were cooled to 4 °C on the autosampler. A linear gradient was applied with acetonitrile + 0.025% FA (A) / water + 0.04% TFA + 0.05% FA (B) (20-50 % A) over a period of 20 min, using an XBRIDGE BEH300 C18 column (3.5 μm, 150 x 2.1 mm) (Waters, France). The source temperature was maintained at 120 °C and the desolvation temperature at 400 °C. The cone voltage and the collision energy were 20 V and 5 V, respectively. HYNIC-D-P8RI starting material was analyzed after solubilization in H_2_O/CH_3_CN (1/4) containing 0.1% FA. ^99^Tc-HYNIC-D-P8RI was prepared as described below with the coligands exchange procedure but with ^99^Tc instead of ^99m^Tc, and it was then analyzed in the same FA buffer. The mass spectra were combined on the peaks at 5.6 min and 6.9 min for HYNIC-D-P8RI and ^99^Tc-HYNIC-D-P8RI samples, respectively. MaxEnt 3 Software (Waters, France) was used for the deconvolution of all mass spectra.

Chemical and radiochemical purities

CP and RCP were determined using a reverse phase high performance liquid chromatography (RP-HPLC) system (Ultimate 3000, Dionex corporation, CA, USA) coupled to a multiwavelength detector (DAD-3000, Dionex) set at 260 nm and a dedicated radio flow monitor (HERM LB 500, Berthold, Germany). An ACE C18 column (150 × 4.6mm, 3 µm, 100 Å; Advanced Chromatography Technologies Ltd, Aberdeen, UK). The flow rate was 1 mL/min with a isocratic mobile phase with 77% C (0.1 % TFA in water) and 23% D (CH_3_CN) over the first 2 min, followed by the linear gradients: 23-50% D (2-20 min) and 50-100% D (20-23 min). The CP and RCP were calculated as the percentage of the total area under the curve at 260 nm and radio-detector, respectively.

Thin Layer chromatography

TLC was performed using a radiochromatograph (MiniGita, Elysia-Raytest, Belgium). Stationary phase was silica gel (ITLC-SG, Agilent technologies, CA, USA) and different mobile phases were employed. Methylethylketone (MEK) was used to determine the amount of free ^99m^TcO_4_^-^ (Rf = 1), CH_3_CN/H_2_O (3/2) for ^99m^Tc-colloid (Rf = 0) and Anticoagulant Citrate Dextrose Solution (ACD-A, Baxter International, IL, USA) to determine non-peptide bound ^99m^Tc-coligands and ^99m^TcO_4_^-^ (Rf = 1).

Radiolabeled peptide purification

Whenever RCP was <94%, the radiolabeled preparation was purified using solid phase extraction (SEP-PAK C18 Plus Light Cartridge, Waters, France). The cartridge was washed with 5 mL ethanol and then equilibrated with 5 mL water. The crude mixture was then loaded, followed by 5 mL of water to wash hydrophilic impurities. Radiolabeled peptide was eluted using either ethanol/H_2_O (9/1) for plasma stability studies or acetonitrile/H_2_O (9/1). The ethanol or acetonitrile were then evaporated under nitrogen flux.

Log P value

500 µL of ^99m^Tc-HYNIC-D-P8RI in PBS (pH 7.2) was added to 500 µL of octanol in a 1.5 mL conical vial. After 15 min of vortexing, the vial was centrifuged at 5000xg for 3 min. A 100 µL aliquot of both phases was collected in triplicate and counted with a gamma counter then log P values were calculated.

Fluorescence microscopy

A fourteen-week-old male Apo E-/- mouse (Charles River, Saint Germain sur l'Arbresle, France) was used. After anesthesia by injection of 1 mL/kg of Ketamine 1000 mg + Xylazine 2% intraperitoneally, the mouse was shaved followed by a 100 µL injection of turpentine oil into the right gastrocnemius muscle. Finally, the mouse was sacrificed at day four for histological analysis after injection of 10 µL of P8RI fluorescent probe (conjugation with 5-Carboxytetramethylrhodamine at the N-ter, custom synthesis by MIMOTOPES). The acquisition was performed in the rhodamine fluorescence channel of an Axio Observer microscope, Zeiss, Germany.

**RESULTS**

**Synthesis of the HYNIC-P8RI**

The synthesis of HYNIC-P8RI was performed by solid phase peptide methodology using Fmoc chemistry. The octapeptide sequence was first assembled and subsequently extended on the N-ter lysine alpha position with the PEG spacer arm and with the HYNIC. After cleavage from the resin and deprotection, the purification yielded HYNIC-P8RI (52% yield, Fig. 1 a). This conjugate was characterized by RP-HPLC (purity 96.3 %, Fig. 1 b), by electrospray ionization mass spectrometry (ESI-MS) and by amino acid analysis, all analyses being consistent with the expected characteristics.
